# Transparent Nanotubular TiO_2_ Photoanodes Grown Directly on FTO Substrates

**DOI:** 10.3390/molecules22050775

**Published:** 2017-05-10

**Authors:** Šárka Paušová, Štěpán Kment, Martin Zlámal, Michal Baudys, Zdeněk Hubička, Josef Krýsa

**Affiliations:** 1 University of Chemistry and Technology Prague, Technická 5, 166 28 Prague 6, Czech Republic; zlamalm@vscht.cz (M.Z.); baudysm@vscht.cz (M.B.); 2 Palacký University, RCPTM, Joint Laboratory of Optics, 17. Listopadu 12, 771 46 Olomouc, Czech Republic; stepan.kment@upol.cz (Š.K.); hubicka@fzu.cz (Z.H.)

**Keywords:** nanotubular, transparent, TiO_2_, sputtered Ti, FTO, anodization, photocurrent

## Abstract

This work describes the preparation of transparent TiO_2_ nanotube (TNT) arrays on fluorine-doped tin oxide (FTO) substrates. An optimized electrolyte composition (0.2 mol dm^−3^ NH_4_F and 4 mol dm^−3^ H_2_O in ethylene glycol) was used for the anodization of Ti films with different thicknesses (from 100 to 1300 nm) sputtered on the FTO glass substrates. For Ti thicknesses 600 nm and higher, anodization resulted in the formation of TNT arrays with an outer nanotube diameter around 180 nm and a wall thickness around 45 nm, while for anodized Ti thicknesses of 100 nm, the produced nanotubes were not well defined. The transmittance in the visible region (λ = 500 nm) varied from 90% for the thinnest TNT array to 65% for the thickest TNT array. For the fabrication of transparent TNT arrays by anodization, the optimal Ti thickness on FTO was around 1000 nm. Such fabricated TNT arrays with a length of 2500 nm exhibit stable photocurrent densities in aqueous electrolytes (~300 µA cm^−2^ at potential 0.5 V vs. Ag/AgCl). The stability of the photocurrent response and a sufficient transparency (≥65%) enables the use of transparent TNT arrays in photoelectrochemical applications when the illumination from the support/semiconductor interface is a necessary condition and the transmitted light can be used for another purpose (photocathode or photochemical reaction in the electrolyte).

## 1. Introduction

With an increasing demand on energy supply, the necessity of using an efficient renewable source of energy is growing. One of the possibilities is the conversion of solar light to electricity or fuel. Since photocatalytic water splitting on TiO_2_ was discovered by Fujishima and Honda [1], great effort has been devoted to the application of TiO_2_ in energy conversion. Although TiO_2_ is a suitable candidate for a photoanode, some major drawbacks need to be overcome, such as the high electron–hole recombination. The use of 1-D nanostructures (nanotubes, nanorods, or nanowires) might be a way of improving the efficiency of electron transport through TiO_2_ film. Nanotubular structures have a mechanical stability that is superior to that of nanorod or nanowire structures. Nanotubular structures of TiO_2_ can be prepared by various methods such as the anodization of Ti substrates [2,3,4,5] or of Ti thin films [6,7,8,9], template synthesis [10], and hydrothermal synthesis [5].

Because of their ability to utilize backside illumination, transparent films of TiO_2_ on a conductive substrate are considered to be very promising photoanodes for either DSSCs [11,12,13] or photoelectrochemical water splitting [14,15]. So far, most transparent TiO_2_ nanotubular arrays have consisted of nanotubes prepared on bulk material and then transferred and attached on a transparent substrate [16,17]. However, the fully transparent nanotubular TiO_2_ arrays prepared directly by the complete anodization of a metallic Ti thin layer deposited on a fluorine-doped tin oxide (FTO) glass substrate would represent the most straightforward solution [6,7,8,9,18,19,20,21,22,23,24]. Nanotubes prepared on FTO glass are mainly tested as photoanodes in DSSCs [7,21,23,24] and not in PEC (photoelectrochemical) water splitting [9,22], so there is not much work dealing with photoelectrochemical measurements in an aqueous phase for TNTs prepared directly on FTO glass. We recently showed that the magnetron sputtering of Ti on an FTO substrate appears to be the most promising technique for the anodic fabrication of transparent TNT arrays [25]. This work was thus devoted to the preparation of TNT arrays via anodic oxidation of sputtered Ti films of different thicknesses and to a comparison of their photo(electro)chemical properties with TNT arrays prepared via anodic oxidation of Ti foil.

## 2. Results and Discussion

### 2.1. Optimizing Electrolyte Composition

**Figure 1:**
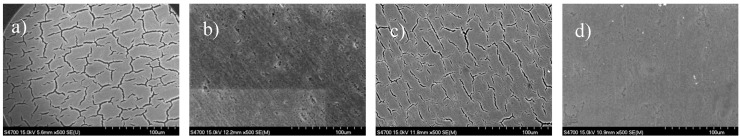
SEM images of TiO_2_ nanotubes (TNTs) prepared by anodization of Ti foil in electrolytes with different water content. Non-annealed: (**a**) 1 mol dm^−3^ H_2_O; (**b**) 2 mol dm^−3^ H_2_O. Annealed at 500 °C; (**c**) 2 mol dm^−3^ H_2_O; (**d**) 4 mol dm^−3^ H_2_O.

Water content in electrolytes has been shown to be a very important parameter [26]. Therefore, this parameter has been studied in detail. Figure 1 shows the surface morphology of TNTs prepared by the anodization of Ti foil in electrolytes with different water concentrations influenced by annealing at 500 °C. Other conditions such as NH_4_F concentration, temperature, applied voltage, and anodization time were kept constant. 

For TNTs prepared with a water content of 1 mol dm^−3^ and lower, cracks appeared even before annealing (Figure 1a). The increase in water concentration to 2 mol dm^−3^ led to a significant decrease in the number of cracks in the TNT array; however, after annealing cracks appeared again in the TNT film (Figure 1c). The optimal amount of water was determined as 4 mol dm^−3^, all films prepared using this electrolyte composition were free of cracks, even after annealing. These results agree well with findings of Tsui et al. [27], who report that a water content around 10% (5.5 mol dm^−3^) results in a well-formed nanotube film with the highest photocurrent density. Additionally, the influence of different NH_4_F concentrations in the 0.1–0.2 mol dm^−3^ range was studied. Resulting layers were similar and no significant influence on their structure was observed, which is in agreement with published results by Acevedo-Pena et al. [28]. Concentrations of 0.2 mol dm^−3^ NH_4_F and 4 mol dm^−3^ H_2_O were thus chosen for further study.

### 2.2. The Growth of TNT Array and Optical Properties

**Figure 2:**
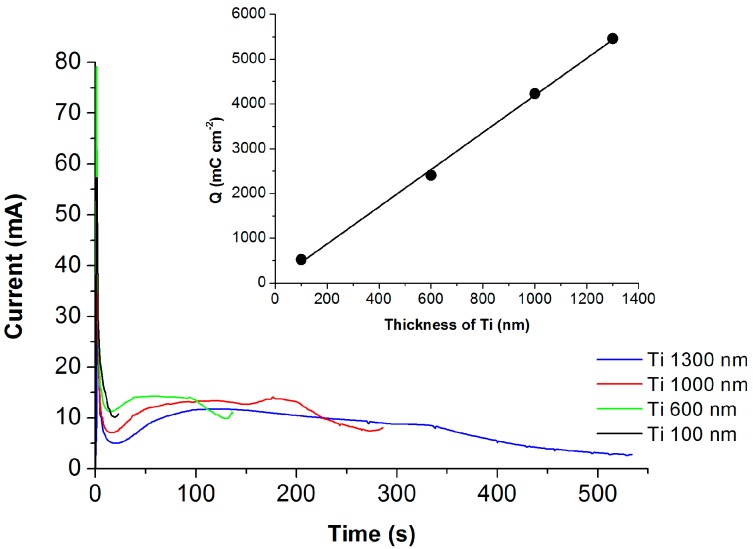
Current density transients during anodization of sputtered Ti films of four different thicknesses to transparent TiO_2_ nanotubes on fluorine-doped tin oxide (FTO) glass. Inset: Passed charge as a function of Ti layer thickness.

Figure 2 shows the current density transient during anodization of a sputtered Ti metal layer of different thicknesses (100, 600, 1000, and 1300 nm) on FTO glass. All current transients show the typical trend of TNT formation. A fast decrease of current corresponds to the formation of compact TiO_2_ followed by a slow increase of current and its stabilization typical of TNT formation. The anodization of Ti films on FTO was terminated when the transparent film was formed. The passed charge depends almost linearly on the Ti film thickness (see the insert in Figure 2). 

**Figure 3:**
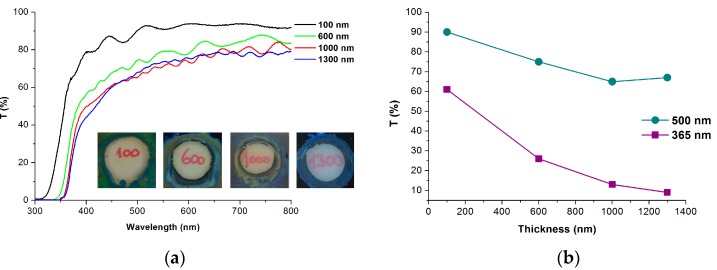
(**a**) UV-Vis spectra of transparent TNT films prepared via the anodization of Ti films of various thicknesses on FTO glass and annealing at 500 °C. Inset: Optical images of anodized Ti films on FTO glass; (**b**) The dependence of T at 365 nm and T at 500 nm (corresponding to annealed TNT films) on the thickness of Ti films on FTO.

The UV-Vis spectra of anodized Ti films of four different thicknesses were corrected on the transmittance of the FTO substrate and are shown in Figure 3a. The transmittance of the TNT film in the whole range of spectra decreases as layer thickness increases—however, the difference between anodized Ti films of thicknesses 1000 and 1300 nm is very small. 

The inserts in Figure 3a shows that all TNT arrays on FTO were transparent. The next step is the evaluation of the transparency in the visible region of solar light and the amount of absorbed light in the UV region. The transparency of the fabricated TNT array is expressed as the transmittance (T) at 500 nm in Figure 3b. This transmission is significantly higher than that in the UV region (90% and 35% for the anodized 100 nm and 1000 nm Ti film, respectively). This means that, even for the thickest TNT; more than 60% of the light in the visible part of the solar spectra is transmitted.

Figure 3b shows the values of T for 365 nm. It is known that the amount of reflection component of the light is not negligible; therefore, the absorbed amount of light cannot be simply obtained as (1 − T). Therefore, for the calculation of absorbed light, we also have to measure the total reflectance (diffuse plus specular) and correct the measured transmittance. This task is possible in the case of single dense layers on a transparent support, as we show for the case of TiO_2_ films of various thicknesses on quartz glass in our previous work [29]. However, in the case of a two-layer structure (TNT arrays on the FTO layer) on a glass support, due to the numerous layer interfaces and the nanostructure character of TNT arrays, the exact subtraction of the reflectance is almost impossible due to light interference. The experimentally obtained reflectance of TNT arrays on FTO was thus only used to estimate the amount of reflected light. For all fabricated TNT arrays with thicknesses of 180–3500 nm, the average value of R in the range from 350 to 800 nm lies in the range 15 ± 5%. This value has been taken for the estimation of absorbed UV light at 365 nm. As expected, the amount of absorbed UV light increases with TNT length (from 25 ± 5% for anodized 100 nm Ti film to 70 ± 5% and 75 ± 5% for anodized 1000 and 1300 nm Ti film).

### 2.3. Crystallinity and Morphology of TNT Arrays

**Figure 4:**
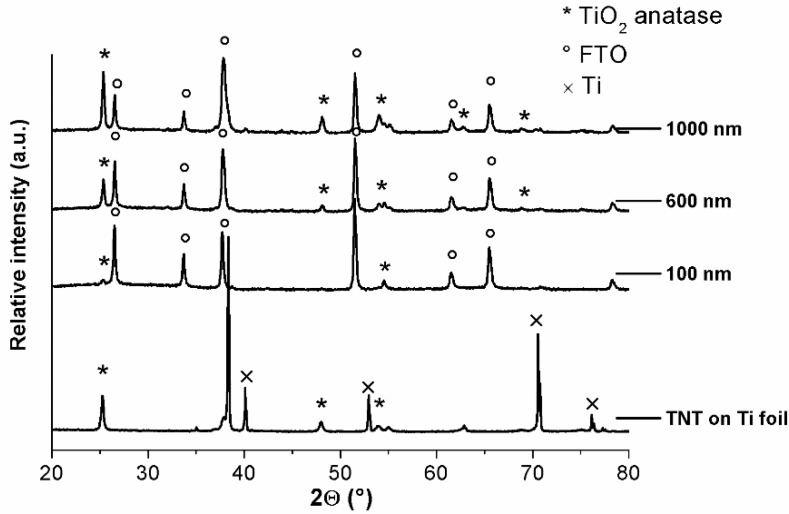
XRD patterns of TNT arrays prepared on Ti foil and on FTO glass and annealed at 500 °C.

Figure 4 shows the diffractograms of the TNT arrays grown on FTO glass substrates. For comparison, the diffractogram for TNTs grown on Ti foil under the same anodization conditions with a passed charge similar to anodization of the 1000 nm Ti film is also shown. After anodization and annealing in an air atmosphere at 500 °C, the TNT arrays on FTO glass and on Ti foil were well crystallized, and the presence of anatase phase was confirmed via the observation of the most intense plane orientations (**110**), (**200**), and (**105**) (Figure 4). There is almost negligible signal corresponding to Ti for anodized Ti films on FTO, implying that Ti was completely oxidized during anodization.

**Figure 5:**
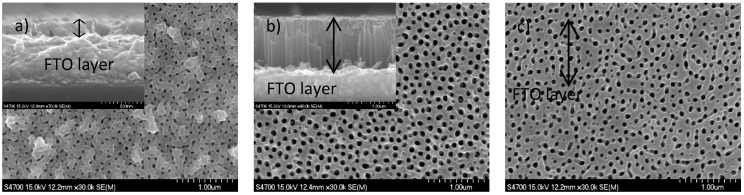
SEM top view and cross-sectional images of TNTs on FTO prepared by the anodization of Ti films of thickness: (**a**) 100 nm; (**b**) 600 nm; and (**c**) 1000 nm and annealed at 500 °C. The thickness of anodized Ti films is marked with a double arrow.

The surface morphology of anodized Ti films was followed by top-view SEM images; the thickness and structure of anodized Ti films was determined from cross-section SEM images (see Figure 5). The structure of the thinnest transparent film was not completely nanotubular—the beginning of the formation of the nanotubular structure is shown in Figure 5a—but in the cross section, the nanotubular structure was not observed. This is due to the very short (25 s) anodization period, which is not sufficient for the development of a proper nanotubular structure. The anodization of thicker Ti films resulted in the fabrication of well-formed nanotubes; however, they were covered with a thin and irregular nanoporous structure. The presence of this top layer has been reported elsewhere and can be, for some purposes, removed by sonification in dilute HCl or by Ar ion sputtering [30].

The anodization of the 100 nm Ti film resulted in the formation of a transparent nanoporous film with a thickness of 180 ± 20 nm. The anodization of the 600 nm and 1000 nm Ti films resulted in the formation of a transparent TNT array with a thickness of 1000 ± 50 nm and 2450 ± 50 nm, respectively. The expansion factor F_ex_ for the anodization of the 100 nm and 600 nm Ti films was in the 1.6–1.8 range. For the 1000 nm Ti film, F_ex_ increased to 2.5. The anodization of the 1300 nm Ti film resulted in the formation of a TNT array with a thickness of 3500 ± 50 nm (not shown in Figure 5) and an expansion factor F_ex_ very similar (2.6) to that for the anodized 1000 nm Ti film.

F_ex_ is highly dependent on electrolyte composition (mainly water content) and applied potential [8]. For the present anodization conditions (a water content of 7 wt %, a potential of 60 V), Albu et al. [8] reported for an evaporated 1000 nm Ti layer on FTO a much lower F_ex_ value (1.7). For a lower water content (3 wt %), Albu et al. [8] reported a value of F_ex_ around 2.6, which was also observed in our recent work [25] but for the anodization of a sputtered Ti film on FTO.

This suggests that, for sputtered Ti films on FTO, the water content is not as critical as it is for evaporated films, and the starting thickness of a sputtered Ti film is more significant. The reason for the increase in the expansion factor with thickness of sputtered Ti film on FTO is not completely clear at the moment. In a detailed previous study, Albu et al. [8] concludes that the variation in the expansion factor can be due to the variation in parameters such as efficiency of oxide growth, chemical composition, density, and porosity of the TiO_2_ nanotubular array. We also think that the influence of the chemical composition, density, and the porosity of Ti film is crucial. In our work, Ti films were grown by magnetron sputtering with emphasis on the adhesion of Ti to FTO. Therefore, the composition and porosity of Ti films close to FTO glass can be different from that of the bulk of Ti films, and this influences the expansion factors of TNTs obtained by anodization of Ti with less thickness, where the different interfacial parts of the Ti films close to FTO represent significant parts of the entire Ti film.

The outer diameter of TNTs for samples prepared from 600 nm, 1000 nm, and 1300 nm Ti films was between 160 and 190 nm, with a wall thickness between 40 and 50 nm. These characteristics differ for samples 100 nm thick; the nanotubes are not that well defined, the wall thickness varies between 40 and 70 nm and the inner diameter is about 50 nm.

### 2.4. Photoelectrochemical Activity

**Figure 6:**
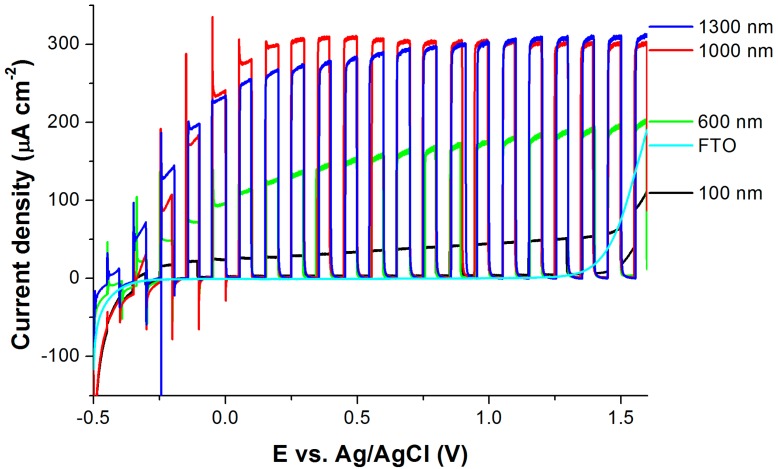
Chopped light polarization curve for TNTs of different anodized Ti thicknesses on FTO glass annealed at 500 °C, electrolytes of 0.1 mol dm^−3^ Na_2_SO_4_.

The photoelectrochemical behavior of anodized films on FTO glass has been compared in aqueous electrolytes with the aim of evaluating the efficiency of PEC water splitting. Figure 6 shows the comparison of chopped light polarization curves under simulated solar light for TNTs prepared from Ti films on FTO glass annealed at 500 °C. The photocurrent onset is around −0.4 V (Ag/AgCl), and at 0 V the photocurrent plateau starts to develop. The photocurrent density in plateau increases as the anodized Ti thicknesses increase from 100 to 1000 nm, but for Ti thicknesses 1000 and 1300 nm, it is almost identical. This is in agreement with the fact that for thicknesses of 1000–1300 nm, the amount of absorbed UV light is very similar (see Figure 3b). The chronoamperometry measurements enabled the evaluation of the photocurrent stability as a function of irradiation time. This was performed on all TNT films on FTO, and the photocurrent density in plateau (at 1 V (Ag/AgCl)) was stable for at least 5 min. Values are shown in Table 1. At least two samples of Ti films with identical thicknesses on FTO were anodized to obtain transparent TNT films. The deviation in the measured photocurrents for each TNT film was lower than 10%. The photocurrent density of the TNT film with a thickness of 2500 nm (prepared from the 1000 nm Ti film) reached 300 µA cm^−2^ at 0.5 V (Ag/AgCl), which is 25 times higher than the value published by Szkoda et al. [9] (although they prepared transparent TNT films from Ti films with a greater thickness (2000 nm). Bai et al. [22] reported a substantially high value of photocurrent density (750 µA cm^−2^ at 0.2 V (Ag/AgCl)) for transparent TNT films. However, the measurement was carried out in 1 mol dm^-3^ Na_2_S electrolytes, which can act as a hole scavenger [31] and thus increase the PEC performance of a TNT layer.

A long test was performed for the anodized Ti film with a thickness of 1000 nm on FTO. The photocurrent density at the beginning of the test was 296 µA/cm^−2^ and after 3 h it decreased to 282 µA cm^−2^ (about 95% of the initial value). The highest decrease was observed after the first 30 min; afterwards, the value of the photocurrent remained almost stable at 282 ± 2 µA cm^−2^. The summarized values of the photocurrents are shown in Table 1. The TNT film on FTO prepared from 1000-nm-thick Ti was compared with TNT films on Ti foil (with a comparable charge passed during anodization). The photocurrent densities obtained by both types of TNT films were similar (about 300 µA cm^−2^ at 1 V against Ag/AgCl). This means that such fabricated transparent TNT arrays can be successfully used in photoelectrochemical applications that require illumination from the support/semiconductor interface.

### 2.5. Photocatalytic Activity

For each TNT array sample annealed at 500 °C, the photoactivity test using Resazurin indicator ink was performed. Resazurin ink works via a photo-reductive mechanism and photocatalytic activity is determined by its color change from blue to pink. The rate of the color change of the ink, measured using digital photography, provides a direct measurement of the photocatalytic activity.

**Figure 7:**
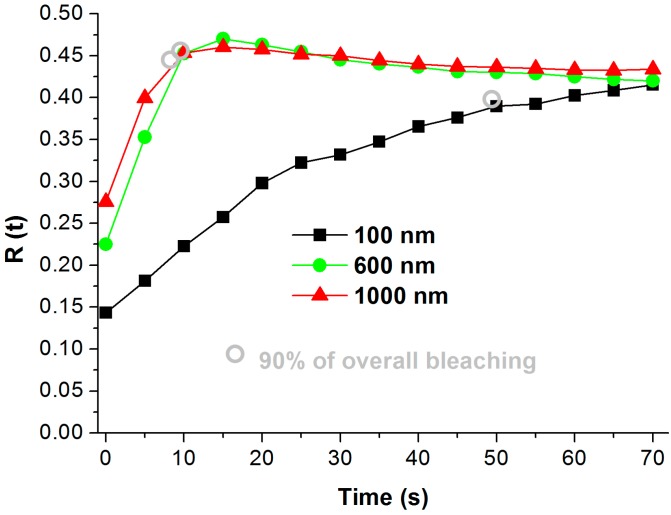
Dependence of R(t) corresponding to Resazurin ink on UV exposition time for TNT arrays of different anodized Ti thicknesses on FTO glass, annealed at 500 °C.

The plot of the R vs. the time of UV exposure for TNTs of various thicknesses is shown in Figure 7. R(t)90 (90% of overall bleaching) was used to calculate the time to bleach 90% of the color, ttb(90) [21] and thus to compare the photocatalytic activity of prepared TNT films. ttb(90) values are marked by gray circles in Figure 6 and shown together with the reciprocal values 1/ttb(90) (proportional to photoactivity) in Table 2. All annealed TNT arrays were photoactive. The photocatalytic activity of TNT arrays on FTO glass significantly increased with an increase in anodized Ti thickness from 100 to 600 nm; however, further increases in the thickness of anodized Ti did not result in an additional photoactivity increase. The ttb90 value for non-anodized Ti foil and non-annealed TNTs was for both samples higher than 420 s, which implies that those samples exhibit about two orders of magnitude lower photoactivity. In the case of Ti foil, the ttb90 value corresponds to the very thin (several nm) film of native TiO_2_ oxide on Ti foil; in the case of non-annealed TNTs, it corresponds to the amorphous TiO_2_.

The fact that the above Ti thickness of 600 nm, the photocatalytic activity of fabricated TNT arrays does not increase, while the photoelectrochemical activity increases to a Ti thickness of 1000 nm, which is rather surprising. There is one possible explanation. The deposited ink film is in contact only with the upper part of the TNT array. The photoactivity of TNTs with thicknesses of 180 and 1000 nm differ by a factor of 5, so photoactivity is proportional to the TNT length and roughly to the exposed surface area of TNT arrays. An increase in TNT length from 1000 to 2500 nm then results in a photoactivity increase of about 10%, which does not correspond to the increase in TNT length. This suggests that the bottom part of the TNTs of a higher thickness (than approximately 1000 nm) is not in contact with ink film and thus cannot be utilized for photocatalytic reactions.

## 3. Experimental Part

### 3.1. TNT Preparation and Characterization

Self-organized TiO_2_ nanotubes (TNTs) were grown via the electrochemical anodization of a Ti foil (purity 99.6%, Advent Research Materials Ltd., Oxford, UK) or of titanium film deposited on FTO.

Titanium films were deposited on FTO substrates (FTO-TCO22-7, Solaronix, Aubonne, Switzerland) via pulsed magnetron sputtering using a pure titanium target. An operating pressure of 0.2 Pa was kept constant during the deposition. The duty cycle of the pulse was 90% and the frequency was 50 kHz. In order to improve the adhesion of titanium, the FTO glass substrate was treated by radio-frequency (RF) plasma before the deposition. The substrate holder worked as an RF electrode connected to the RF power supply working at 13.6 MHz. A gas mixture of Ar–O_2_ was used for this purpose, with a pressure of 10 Pa in the reactor chamber. The deposition process followed immediately after RF plasma treatment without the interruption of a vacuum in the reactor chamber. The deposition time differed according to the prepared Ti film thickness: 100 nm—3.5 min, 600 nm—21 min, 1000 nm—35 min, 1300 nm—45.5 min; deposition speed was the same for all samples.

The Ti foil was chemically polished in a mixture of HF and HNO_3_ and then washed in ethanol and acetone in an ultrasonic bath. The Ti films on FTO glass were just washed with ethanol. TNTs were grown at 60 V using a power source (STATRON 3253.3, Statron AG, Mägenwil, Switzerland) in a two-electrode configuration with a counter electrode made of platinum in electrolytes containing 0.1–0.2 mol dm^−3^ NH_4_F + 0.5–8 mol dm^−3^ H_2_O in ethylene glycol. After the anodization process, the samples were washed in ethanol and then dried in a nitrogen stream. To transfer amorphous TiO_2_ nanotubes to a crystalline anatase structure, anodized samples were annealed at 500 °C for 1 h in air using a cylindrical furnace (Clasic CLARE 4.0, CLASIC CZ Ltd., Řevnice, Czech Republic) with a temperature increase of 5 °C min^−1^.

### 3.2. Characterization Methods

The titania nanotubes produced were characterized by XRD (PANanalytical X´Pert PRO, Cu tube, 1D XCelerator detector, PANanalytical B.V., Almelo, Netherlands), SEM (Hitachi SEM S-4700, Hitachi, Tokyo, Japan), and UV-Vis spectroscopy (Varian CARY 100 with integrating sphere DRA-CA-30I and 8 reflectance geometry, Varian, Palo Alto, CA, USA).

### 3.3. Photocatalytic Activity

Photocatalytic activity was determined using degradation of Resazurin in model ink. Photocatalytic activity indicator inks work on the basis that (i) the ink film is deposited onto the surface of the photocatalytic film under test; (ii) upon ultra-band gap illumination of the underlying photocatalyst, the photogenerated holes oxidize a sacrificial electron donor (such as glycerol), which is present in the ink. The photogenerated electrons are then free to reduce the redox dye present in the ink and in doing so change the color of the ink. The redox dye is chosen so that it is readily and irreversibly reduced by the photogenerated electrons and that the associated color change is striking. A typical ink consisted of 10 g of 1.5 wt % of hydroxyethyl cellulose, 1 g of glycerol, 20 mg of polysorbate 20 surfactant, and 10 mg of the Resazurin dye [32]. The amount of dye in the ink was chosen based on previous experience with an aim to achieve decolorization times in the range from 1–90 min [33,34]. Ink was coated on a photocatalytically active surface using a paintbrush. After application, the ink-coated sample was dried for 1 h in the dark and then exposed to UVA light from a black light (BL) lamp (λ_max_(emission) = 352 nm; irradiance: 2 mW/cm^2^). Throughout the irradiation, digital images of the ink-coated samples were periodically recorded using a handheld document scanner (CopyCat). The central part of the ink-coated digital image (with minimal dpi 300) of each sample was then analyzed in terms of its *RGB* values (*RGB*(*red*)*_t_*, *RGB*(*green*)*_t_*, and *RGB*(*blue*)*_t_*) using a free graphical software package, ImageJ. The normalized color component *R*(*t*) value for red was calculated according to Equation (1):

(1) $$R\left(t\right)=\frac{RGB{\left(red\right)}_{t}}{RGB{\left(red\right)}_{t}+RGB{\left(green\right)}_{t}+RGB{\left(bleu\right)}_{t}}.$$

The present method of ink deposition (paintbrush) was different from that in our previous work [29,32], where a 24 micron K-bar was used to obtain the same thickness and homogeneous distribution of the ink film on smooth photocatalytic surfaces. The amount of deposited ink could be monitored by the measurement of the initial normalized color component *R*(*t* = 0) value. This value was for all tested TNT samples in the 0.14–0.27 range, which was found to be sufficient for the achievement of the reproduction (20% deviation) of the amount of deposited ink on the TNT arrays of various lengths.

### 3.4. Photoelectrochemical Activity

A 150 W Xe arc lamp (Newport) with an AM1.5G filter (100 mW cm^−2^) was used for the photoelectrochemical characterization of TiO_2_ nanotubes. Photocurrents were acquired using a photoelectrochemical cell in aqueous 0.1 mol/dm^−3^ Na_2_SO_4_ electrolytes using a conventional three-electrode configuration (a Pt counter electrode, an Ag/AgCl reference electrode, and the TNT array as the working electrode). The photocurrent was recorded versus the applied potential using a potentiostat interfaced to a computer. The linear voltammetry of prepared layers was measured with a sweep rate of 5 mV/s^−1^ while being periodically illuminated (5 s light/5 s dark). Chronoamperometry measurement was performed at 0.5 V against an Ag/AgCl reference electrode (1.45 V vs. RHE) under continuous illumination.

## 4. Conclusions

The insufficient water amount in the electrolytes led to a high amount of cracks in the TNT layer, so the electrolyte composition had to be optimized. The anodization of Ti films with thicknesses of 100–1300 nm on FTO resulted in transparent TNT arrays; the observed expansion factor depended on the Ti thickness and varied from 1.8 for Ti thicknesses lower than ≈600 nm to 2.5 for Ti thicknesses higher than ≈1000 nm. The transmittance in the visible field (500 nm) varied from 90% for the thinnest TNT array to 65% for the thickest TNT array. The photocatalytic activity was successfully demonstrated by the decolorization of Rz ink. Fabricated TNT arrays on FTO glass showed an increase in photocurrent density as TNT length increased, but for TNT lengths higher than 2500 nm, the photocurrent was fairly constant. For the fabrication of transparent TNT arrays, the optimal Ti thickness is around 1000 nm. This is due to the absorption of a significant amount of UV light (≥70%) and a sufficient transparency (≥65%), which enables their use in photoelectrochemical applications when the illumination from the support/semiconductor interface is a necessary condition.

## Figures and Tables

**Table 1:** Main characteristics of all prepared samples.

Name of Sample	Ti Film Thickness (nm)	Anodization Time (s)	Passed Charge (mC cm^−2^)	TNT Thickness (nm)	Photocurrent Density * (µA cm^−2^)
100 nm	100	25	524	180 ± 20	43 ± 2
600 nm	600	138	2404	1000 ± 50	196 ± 5
1000 nm	1000	290	4226	2450 ± 50	300 ± 5
1300 nm	1300	538	5460	3500 ± 50	294 ± 5
Ti foil	-	887	4155	-	315 ± 5

* Results obtained by chronoamperometry measurement at 1 V vs. Ag/AgCl in Na_2_SO_4_ electrolytes.

**Table 2:** ttb(90) values of Resazurin reduction for various anodized Ti thickness.

Thickness of Ti on FTO	TNT Thickness	ttb(90) (s)	1/ttb (90) (s^−1^)
100 nm	180 nm	50	0.020
600 nm	1000 nm	10	0.100
1000 nm	2500 nm	9	0.111
Ti foil	-	≥420	≤0.0024
non-annealed TNT	-	≥420	≤0.0024
